# Psychosocial perception of adults with onychomycosis: a blinded, controlled comparison of 1,017 adult Hong Kong residents with or without onychomycosis

**DOI:** 10.1186/1751-0759-8-15

**Published:** 2014-07-15

**Authors:** Henry HL Chan, Emma T Wong, Chi Keung Yeung

**Affiliations:** 1 Division of Dermatology, Department of Medicine, The University of Hong Kong, 4/F Professorial Block, Queen Mary Hospital, 102 Pokfulam Road, Hong Kong, Hong Kong; 2 4B, Valiant Commercial Building, 22-24 Prat Avenue, TsimShaTsui, Kowloon, Hong Kong

**Keywords:** Onychomycosis, Hong Kong, Perception, Fungal nail infection, Psychosocial

## Abstract

**Background:**

A survey was conducted amongst 1,017 Hong Kong residents ages 18 and over to determine their knowledge of fungal nail infections (onychomycosis) and the psychosocial impact of the disease on the relationships, social lives and careers of sufferers.

**Methods:**

The Fungal Nail Perception Survey was conducted by email and online between May 29th and June 10th, 2013. Participants were shown three photographs of people with and without onychomycosis of the toenails. Respondents were asked ten questions (repeated for each picture) to ascertain their perceptions of the people in the pictures. Questions were related to perceptions around the ability of sufferers and non-sufferers to form relationships with others, social activities of sufferers and non-sufferers, perceptions of the effect of the disease on the potential for career success, and awareness of fungal nail disease and health. The sub-population who themselves suffered from onychomycosis were asked about self-perception as well as their perception of others with onychomycosis.

**Results:**

Compared with non-sufferers, survey respondents perceived those with onychomycosis as less likely to be able to form good relationships. They also indicated that they would be more likely to exclude sufferers than non-sufferers from social activities and that they would be more likely to feel uncomfortable when sitting or standing beside an infected person than beside an uninfected person. Respondents perceived people with onychomycosis to be less able to perform well in their chosen career than with someone without onychomycosis. Interestingly, those respondents who themselves were infected felt socially excluded, upset and embarrassed by their infection.

**Conclusions:**

Onychomycosis may lead to stigmatization and social exclusion. Misconceptions of onychomycosis are high and education about the disease needs to be improved. Early recognition and treatment of the disease is essential to avoid complications and improve treatment outcomes, which would lead to reduced psychosocial impact on those with fungal nail infections.

## Background

Nail care and adornment is an established practice in many societies that contributes to an elevation in personal appraisal by others and to improvements in confidence and self-image in women and men [1]. Conversely, onychomycosis of the finger and toe nails, commonly known as fungal nail infection, impacts the quality of life of sufferers.

Much is known about the pathogenesis and treatment of onychomycosis, but the literature on the perception of the condition amongst sufferers, their family, friends, or strangers is sparse. A recent survey conducted in Hong Kong has revealed the unforeseen potential impact of having fungal nail infections at work and on relationships, together with the frequent over-reaction to and misconception of the disease in social situations.

### Epidemiology

Onychomycosis is one of the most common dermatological infections worldwide. In Europe, a foot disease survey estimated the prevalence to be 26.8% [2]. In Hong Kong, the estimated prevalence of toenail onychomycosis in a similar survey was 21% [3]. In a study of 2,382 infected nail samples conducted by the Department of Health in Hong Kong, the prevalence of onychomycosis was higher in men compared to women in the <19 and >50 years age groups, while women in the 20–50 years age group were affected more [4].

### Causes

Toenail onychomycosis is most commonly caused by dermatophytes; *Trichophyton rubrum* being the predominant causative agent in the USA [5], Europe [6] and Hong Kong [4]. A retrospective survey in Hong Kong over a period of 8 years showed dermatophytes in 66.7% and yeasts in 33.3% in the nail culture from patients with onychomycosis of the toenails [4].

### Factors contributing to the development of onychomycosis

A number of factors can contribute to the development of onychomycosis. These include lifestyle habits such as wearing occlusive footwear, smoking and regular swimming. Onychomycosis is not air transmitted and it is often picked up by walking barefoot on damp floors in communal areas such as gyms, locker rooms, saunas and swimming pools [7,8]. Underlying conditions such as *tinea pedis* (athlete’s foot), nail damage and nail psoriasis can contribute to risk, as do characteristics such as age and obesity. A number of underlying morbidities, such as diabetes [7], cancer [7,8], immunodeficiency [9] or peripheral arterial disease (PAD) [10] increase susceptibility to the infection. Direct contact with other persons, such as family members with onychomycosis, is a risk factor [7]. An inherited genetic predisposition to infection has also been identified [11].

### Clinical features of disease

The initial symptoms of onychomycosis are relatively minor and include nail discoloration, thickening of the nail in response to infection, onycholysis (detachment of the nail from the nail bed), pain and discomfort. Discoloration is often the first symptom and the nail may become yellow-white, but also brown, orange, of black depending on the microorganism responsible. The nail becomes brittle and breaks easily. Pain and discomfort sometimes result from inflammation and pressure from the thickened nail plate [12,13].

Onychomycosis is traditionally classified according to the position of the infection within the nail plate or nail bed. There are distinct classifications based on nail plate pattern changes; distal and lateral subungual (DLSO), superficial, endonyx, proximal subungual, mixed, totally dystrophic, and secondary onychomycosis [13]. Of these, DLSO is the most common type of onychomycosis, affecting males and females in equal measure. In this type, the infection progresses from the lateral or distal undersurface of the nail plate towards the proximal nail fold. Onychomycosis most frequently affects one or two toenails.

### Diagnosis and treatment of onychomycosis

Diagnosis requires a combination of clinical examination for appearance of the nail and the number of nails involved and laboratory examination for identification of the causative organisms. Prompt attention is important, as is differential diagnosis to avoid treatment failure and complications. Treatment depends on the classification and extent of disease. Both topical and oral antifungal agents are available for the treatment of fungal nail infection [14]. A partial removal of thickened, infected nail plate (nail avulsion) can be performed. Total nail avulsion is sometimes necessary for the most persistent and serious cases. There are three main options to treat fungal nail infections: topical antifungals, systemic antifungals and nail plate removal. Topical treatments include ointments, creams and lacquers, usually containing an antifungal drug. Lacquer formulations increase penetration into nail plates leading to an increase in the duration of contact with the antifungal. They are also easy to use. Systemic treatments with antifungal agents are frequently preferred as they are the most effective. However, they may also cause side effects, e.g. interactions with other medications or causing gastrointestinal effects [15]. Partial nail avulsion (either chemical with urea or mechanical) or nail abrasion drastically reduces fungal load and may increase the penetration of topical treatments. Complete avulsion removes the thickened nail plate, but is normally recommended only as a last resort where other treatments have failed, as there is a risk of nail dystrophy with regrowth [16].

### Impact of onychomycosis

If onychomycosis is left untreated, there is a risk of more serious infections and complications developing in those with concomitant diabetes [17] or impaired immunity [8]. There is also an increased risk of the development of bacterial cellulitis [18]. Recent evidence has revealed that onychomycosis may be an early indicator of the onset of PAD [10]. Ignoring infected nails or treating them improperly contributes to an increased risk of complete destruction of the nail and further spread of the fungal infection to surrounding skin, other nails and other parts of the body via auto-inoculation [19]. The risk of transmission of fungal foot infection within the community is also greater. Those persons with type 2 diabetes, the immunocompromised and people with PAD are at particular risk of onychomycosis and severe complications [9,17,18].

### Quality of life

There is already much evidence outlining the impact of onychomycosis on quality of life [20-22]. This may be physical, such as pain, a burning sensation or discomfort that leads to a functional impact on the patient [23]. There is also an emotional impact contributing to an overall negative psychosocial effect. Patients often feel embarrassed and stigmatized [22,24,25].

This manuscript describes the results of a survey of 1,017 representative residents of Hong Kong. It is important to understand how onychomycosis impacts the lives of these residents on a daily basis to determine whether there is a need for further education and understanding of onychomycosis. The survey was designed to determine the psychosocial impact of onychomycosis and to better understand the perception of sufferers from the point of view of fellow sufferers as well as non-sufferers. The results reveal that the psychosocial impact of onychomycosishas the potential to be more wide-ranging and serious than might first be understood for such a simple disease.

## Methods

The objectives of this study were to assess whether onychomycosis changes how others perceive people affected by the disease, their knowledge of onychomycosis and to quantify the social impacts of untreated fungal nail infections. This survey included participants with and without onychomycosis.

**Figure 1:**
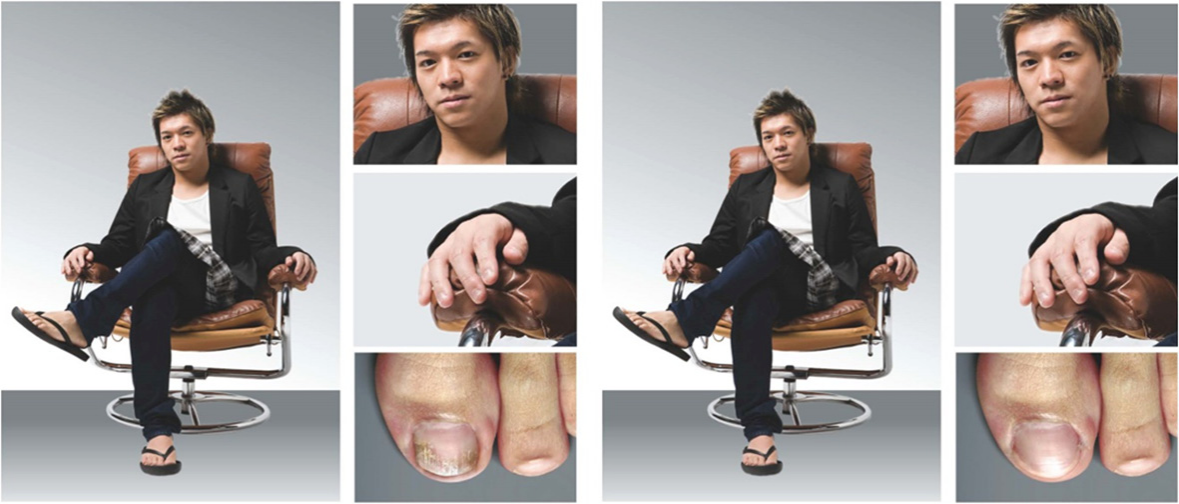
Examples of photographs shown to participants in the Fungal Nail Perception Survey.

The Fungal Nail Perception Survey was conducted between May 29th and June 10th, 2013 among nationally representative Hong Kong residents aged 18 years and over using an email invitation and an online survey. Informed consent was obtained from the respondents for the publication of the findings and no images of the patients were used in this study. Quotas were set to ensure reliable and accurate representation of the entire adult Hong Kong population. Respondents were given the option to take the survey in either English or Chinese. This online survey on the public perception was carried out in compliance with the Helsinki Declaration.As part of the survey, each respondent answered six screening questions. Screening was performed to exclude candidates younger than 18 years old and anyone involved in the marketing industry or market research. Those who passed selection were presented with three whole body pictures of men or women with or without fungal nail infections on their toenails. The toenails were visible in all cases [Figure 1]. The pictures showed people with DLSO infection, as this is the most common form of onychomycosis and most likely to be encountered in daily life. Each picture included full pictures of each individual’s body, as well as close-up shots of the person’s face, fingers, and toenails. In total, the survey consisted of 12 pictures – 50% male and 50% female with one non-infected and one infected image for each of the six separate models. Each respondent reacted to three randomly selected pictures, with the only condition being that respondents view at least one picture showing a healthy person and at least one picture showing someone with an onychomycosis infection. This design allowed the respondents to focus in-depth on specific stimuli.

Upon viewing each picture, respondents were asked ten questions per picture to ascertain their perceptions of the people in the pictures. Questions were related to perceptions about the ability of sufferers to form relationships with others, their social activities (from the perspectives of sufferers and non-sufferers), perceptions about the effect of the disease on potential career success, and awareness of fungal nail disease and health. Respondents were also asked seven questions that did not relate to the pictures. The sub-population who themselves suffered from onychomycosis were asked about self-perception as well as their perception of others with onychomycosis.

Survey results from any sample are subject to sampling variation. The magnitude of the variation is measurable and is affected by the number of interviews and the level of the percentages expressing the results. All comparisons reported in this analysis were tested for statistical significance.

In this study, the chances were 95 in 100 that a survey result did not vary by plus or minus 3.1 percentage points from the result that would be obtained if interviews had been conducted with all persons in the universe represented by the sample. The margin of error for the subgroup of sufferers was slightly higher: plus or minus 7.7 percentage points from the results.

## Results

There were 1,017 people surveyed; 519 (51%) were female and 498 (49%) were male. There were 466 (46%) aged under 40 years, and 551 (54%) aged 40 years and over. Within this population, 163 (16%) admitted to currently having a fungal nail infection.

### Personal relationships

**Figure 2:**
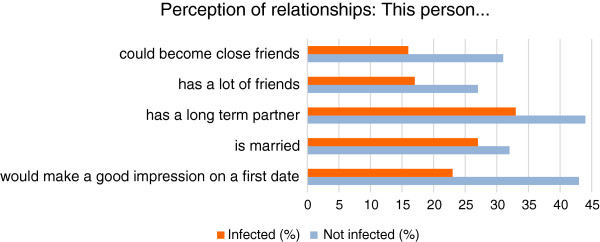
Perceptions about ability to develop relationships with infected and uninfected people.

Based on reviewing the photographs, respondents were asked to rate the ability of those with onychomycosis to develop relationships compared to those with no infection. The results showed that the respondents were almost twice as likely to form close friendships with someone who did not have a fungal nail infection than with someone with an infection (30% vs. 16%, respectively).Respondents also felt that those with visible onychomycosis were unlikely to have a lot of friends (27% vs. 17%),would be less likely to have a significant other (32% vs. 44%) or be married (27% vs. 34%),or make a good impression on a first date (24% vs. 43%) compared with uninfected persons [Figure 2].

### Social activities

**Figure 3:**
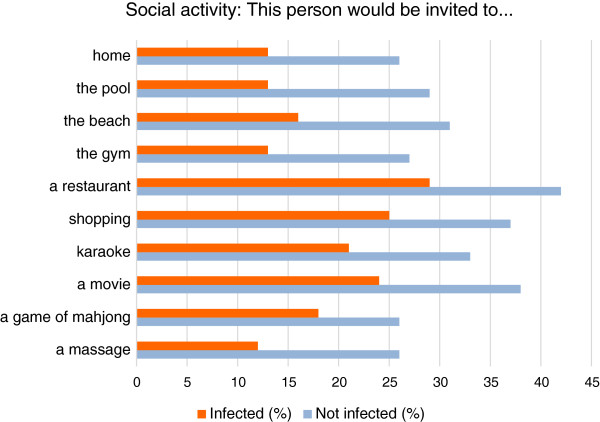
People with onychomycosis are less likely to be invited to social activities.

Respondents were asked how likely or unlikely they would be to interact with infected and uninfected people in various social situations. Infected people were less likely to be invited to the respondent’s home (13% vs. 26%), a swimming pool (13% vs. 29%), the beach (15% vs. 31%), their gym (13% vs. 27%), restaurants (30% vs. 42%), a shopping mall (25% vs. 37%), a Karaokelounge (22% vs. 33%), a movie (24% vs. 38%), a game of mah-jong (18%vs. 26%) or to get a back or foot massage (12% vs. 26%) compared with those who appeared to be uninfected [Figure 3].

### Health

**Figure 4:**
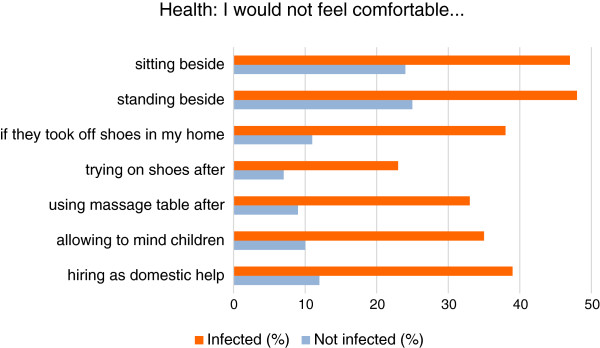
People avoid sufferers of onychomycosis.

The survey assessed perceptions of health and the risk of infection from sufferers of onychomycosis [Figure 4]. Respondents were less likely to feel comfortable sitting (24% vs. 47%) or standing (25% vs. 48%) next to visibly infected people compared with an uninfected person. They were also less likely to feel comfortable about having an infected person remove their shoes in their homes (11% vs. 38%), about trying on a pair of shoes (7% vs. 23%) or using a massage table after an infected person (9 % vs. 33%). Respondents who were parents were also less likely to feel comfortable having an infected person look after their children (10% vs. 35%). Responses showed that people would be less likely to be comfortable hiring an infected person as their domestic helper compared with an uninfected person (12% vs. 39%).

### Personal qualities

The respondents were asked to choose the word they felt would best describe their impressions of an infected individual who used the gym, pool or the beach. Respondents were more likely to perceive those with a visible fungal nail condition as selfish (22% vs. 13%) and less likely to perceive them as friendly (32% vs. 47%) and polite (29% vs. 45%) than those without infection.

### Work place performance

**Figure 5:**
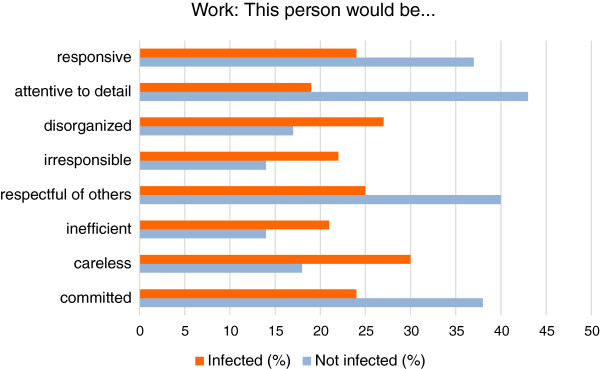
Perceptions of attitudes and activity at work.

The respondents felt that those with visible onychomycosis were more likely to be careless (30% vs. 18%), irresponsible (22% vs. 14%), disorganized (27% vs. 17%), and inefficient (21% vs. 14%) in a work setting compared to those with no infection. Those with onychomycosis were less likely to be described as responsive (24% vs. 37%), attentive to detail (19% vs. 43%), respectful of others (25% vs. 40%), and committed to their jobs (24% vs. 38%) [Figure 5].

### Career

**Figure 6:**
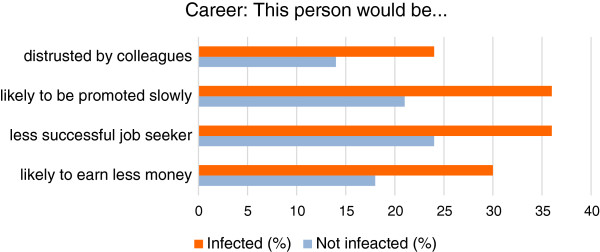
Perceptions of career progression in infected persons.

The respondents felt that, compared to those who were not infected, persons with visible fungal nail infections were more likely to be distrusted by colleagues (24% vs. 14%) and would be promoted more slowly than their peers(36% vs. 21%); they would have more difficulty in getting a job(36% vs. 24%)and would earn less money(30% vs. 18%) [Figure 6].

### Personal hygiene

The respondents were asked to comment on their perceptions of the personal hygiene of persons with and without apparent fungal nail infection. The results indicated that sufferers are more likely to be perceived as washing their feet less often (7% vs. 1%)and as having poor hygiene compared with those without fungal nail infection (38% vs. 7%). By contrast, 52% of the respondents felt that those without fungal nail infection had very good hygiene compared with only 7% of the patients with onychomycosis.

### Knowledge of the disease

The respondents were asked how likely it was that the person in each picture had a contagious medical condition, a non-contagious medical condition or had no medical condition. Exactly 37% of the respondents felt that someone with onychomycosis had a contagious medical condition, while 33% felt that the infected person had a non-contagious medical condition, and 11% felt that the person did not have a medical condition. Conversely, of respondents shown a picture of someone without onychomycosis, 8% felt that the person had a contagious medical condition; while 15% and 30% felt the person had a non-contagious medical condition or did not have a medical condition, respectively.

There were wide-ranging misconceptions around the disease, with over a fifth of the respondents (23%) feeling that fungal nail infections can be caused by long-term use of nail polish, rising to 29% among females. There were misperceptions of treatment, with just over one in ten thinking that natural sunshine could cure the infection (11%) or that infection was a natural part of the aging process (11%). Over one quarter (26%) felt that disinfectant solutions such as vinegar or alcohol were effective treatments, while 14% of the respondents thought the only cure was complete removal of the nail.

When specifically asked about treatment, 60% of the respondents felt that fungal nail infection could be effectively treated with prescription creams, ointments or nail lacquers. Furthermore, the respondents thought that antibiotics (42%), prescription pills (42%), foot or shoe sprays (35%), over-the-counter medicated creams/ointments/lacquers (29%), Chinese herbal tinctures (24%), over-the-counter medication (18%), laser treatment (17%) and nail removal (15%) were effective treatments.

The respondents were clear about certain causes of fungal infection, but harboured misunderstandings and a lack of knowledge of others. For example, 61% of the respondents knew that wearing shoes that make feet moist or sweaty may cause fungal infection, while 59%, 47% and 54% respectively knew that athlete’s foot, humid climates or a weakened immune system may be a predisposing factor, contributing to the condition. However, 30% of the participants said that getting a manicure or pedicure and 29% said that getting a gel nail could predispose them to infection. Only 28% of the participants knew that getting minor nail injuries could predispose them to infection.

General knowledge of the disease was mixed. While 61% of the people felt that they knew what the disease looked like, only 40% were confident that they knew the symptoms of the disease and only 33% admitted to knowing the causes and behaviors that lead to a risk of infection. More than one in ten respondents (13%) admitted knowing nothing about the disease.

### Sufferers

Almost half (47%) of those with onychomycosis admitted that they were sometimes upset by the appearance of their nails. Almost two in five sufferers (38%) sometimes felt that others would see them as unattractive. Almost a third (31%) had noticed others staring at their nails and felt that others did not want to touch them because of their nails (29%) or that others avoided them because of their nails (28%). Many sufferers (33%) felt they had been the subject of unpleasant comments because of their nails.

## Discussion

This survey conducted in Hong Kong revealed many interesting and previously unrecognized misconceptions regarding onychomycosis from the perspective of people with and without the disease. The results contribute to the knowledge of the medical and psychosocial impact of the disease in the community.

Essentially, the survey exposed that there is an exaggerated avoidance of persons with onychomycosis; people are more likely to be negatively judgemental towards sufferers than sympathetic or understanding. Respondents incorrectly linked onychomycosis infection with personal qualities, such as personal habits and lower levels of hygiene. This was translated into nail infections being associated with poor social skills and inability to form close personal relationships: Many respondents said that they would be uncomfortable standing or sitting next to infected persons or inviting them to social activities, even in situations when there is no risk of infection.

Stigmatization of onychomycosis sufferers was not confined to casual social situations. Large numbers of people also reported negative perceptions towards sufferers in the workplace, viewing sufferers as less likely to be promoted, likely to earn less and more likely to be inattentive to detail, inefficient, irresponsible, careless, disorganized and less committed. Clearly these judgments of a person’s qualities may potentially result in a significant negative impact on career prospects.

In the 16% of the study population who identified themselves as onychomycosis sufferers, the survey confirmed the results of prior quality of life studies on the negative psychological, social and occupational impact of onychomycosis. As in these prior studies, persons with onychomycosis in Hong Kong tended to fear the spreading of their disease or to feel embarrassed by their nails and were aware of being stigmatized as a result of the infection, resulting in an unwillingness to engage in social activity or leisure pursuits [22,25-28].

**Figure 7:**
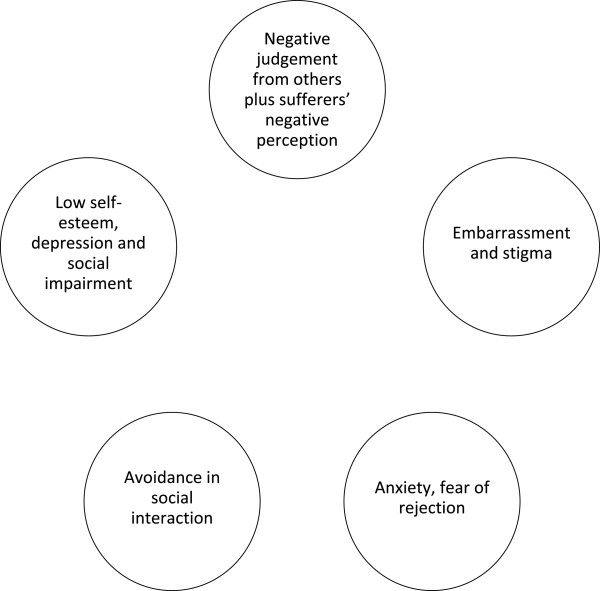
The cycle of psychosocial impact.

This survey shows that while the medical impact of onychomycosis is relatively minor for most people, the psychosocial impact of disease can be tremendous and similar to that of more serious diseases [26-29]. The resulting low self-esteem, depression and social impairment attract further negative judgement and so the vicious cycle goes on [Figure 7].

While many misconceptions contribute to the stigmatization of sufferers, others are actually hazardous to patients and the community. This study showed that not only do many people misunderstand the causes of disease and how it is transmitted, but that many are confused about effective and ineffective treatments for onychomycosis. Misdiagnosis also delays appropriate treatment, contributing to the increased risks associated with infection [19]. For this reason, it is advisable that nail conditions be examined early by a doctor who is qualified to make a correct diagnosis.

Despite the wide-ranging array of effective treatments, over a quarter (26%) of the respondents felt that treating infections with vinegar and disinfectant was sufficient, when in fact nail fungi are resistant to these compounds. Furthermore, over two-fifths of the respondents incorrectly believed that antibiotics were an effective treatment, although only antifungal agents are capable of treating fungal infections.

It is clear from the results of this survey and other studies that onychomycosis can have severe psychosocial impact on sufferers. It is also clear that a misunderstanding of the causes of and treatments for the disease leads to unnecessary and extreme isolation of sufferers. Such negative judgements and perceptions could potentially be avoided if sufferers and non-sufferers had a better understanding and awareness of the disease. Education about onychomycosis is fundamental towards shifting attitudes and dispelling myths and misconceptions. Patients need to be made more aware of the importance of seeing a suitable medical professional as soon as the symptoms of infection appear. They also need to understand that there are effective treatments for the disease and that leaving the disease untreated or inappropriately treating nails can lead to a spread of the infection, complications and potential stigmatisation.

Likewise, general education of the population is needed to show that the development of onychomycosis is not due to poor hygiene and that transmission of the disease is highly unlikely in many social situations. As such, people with onychomycosis do not need to be excluded from social events and should not be stigmatized.

## Conclusions

There is a need for early recognition of onychomycosis and early treatment to restore a good quality of life, maintain social acceptance, and to avoid unnecessarily difficult treatments and medical complications. It is vital to improve education amongst the public as to the nature of onychomycosis, transmission of infection and treatment if we are to dispel myths and misconceptions surrounding onychomycosis. This in turn will lead to reduced psychosocial impact on those with fungal nail infections.

## Abbreviations

DLSO: Distal and lateral subungual; PAD: Peripheral arterial disease.

## Competing interests

Dr Henry Chan declares that: he has acted as a paid consultant to Access Business Group International and Cutera Inc; he has acted as a paid advisor/chairman of advisory board to Basis Medical Technologies Inc, Syneron Medical Ltd, and Lumenis Ltd; he has received fees and honoraria and free and/or discounted access to equipment to conduct clinical trials for Cynosure Inc, Quanta System (Italy), Syneron Medical Ltd (USA), UIthera (USA) and Zeltiq Aesthetics (USA); he holds stock in Basis Medical Technologies Inc, Lumenis Ltd, Solta Medical Inc, and Syneron Medical Ltd; he receives allowances and/or discounts on purchases from Cutera Inc, Lumenis Ltd, and Solta Medical Inc. Dr Chan also receives royalties from McGraw-Hill Companies.

Emma T Wong has no competing interests to declare.

Chi Keung Yeung has no competing interests to declare.

## Authors’ contributions

All of the authors were involved in the development of the survey questions, and all authors actively participated in the development and revision of all content of this manuscript with final approval.
